# Evaluation of Fruit Bagging as a Pest Management Option for Direct Pests of Apple

**DOI:** 10.3390/insects9040178

**Published:** 2018-12-01

**Authors:** Daniel L. Frank

**Affiliations:** Extension Service, Agriculture and Natural Resources Unit, West Virginia University, Morgantown, WV 26506, USA; dlfrank@mail.wvu.edu; Tel.: +01-304-293-8835

**Keywords:** IPM, mechanical control, *Halyomorpha halys*, *Cydia pomonella*, *Venturia inaequalis*, sooty blotch and flyspeck

## Abstract

Bagging fruit with plastic, paper, and two-layer commercial bags was evaluated for control of insect pests and diseases in an experimental apple orchard planted with ‘Red Delicious’ trees. Results from fruit damage evaluations at harvest showed that bagging significantly reduced fruit damage from direct apple pests compared with non-bagged control plots, and generally provided similar levels of fruit protection when compared with a conventional pesticide spray program. Of the three bagging materials evaluated, plastic bags provided numerically higher levels of fruit protection from insect pests, and two-layer commercial bags provided numerically higher levels of fruit protection from fruit diseases. Fruit quality as measured by percentage Brix was higher in non-bagged control plots than all other treatment plots. Fruit quality as measured by fruit diameter was not significantly different among treatments. Plastic and two-layer commercial bags generally required less time to secure around apple fruit than paper bags. The proportion of bags that remained on fruit until harvest ranged from 0.54–0.71 (commercial bags), 0.64–0.82 (plastic bags), and 0.32–0.60 (paper bags), depending on the year.

## 1. Introduction

Apples are an important specialty crop grown in West Virginia. Although the state produced over 42.6 million kilograms of apples in 2017 [1], the rising costs associated with preventing pest related problems have threatened the value of many West Virginia apples. In the Northeastern United States, apple growers may contend with more than 50 direct and indirect arthropod pests, and 20 plant diseases [2]. Because of favorable pest conditions in West Virginia and elsewhere in the Northeast, commercial apple orchards must be intensively managed, often with frequent pesticide applications. Some estimates suggest that Northeastern apple growers may spend 24–30% percent of their production costs on pesticides [3]. 

Currently, many apple growers in West Virginia have expressed interest in reducing pesticide use in orchards. The primary reasons include increasing demand from consumers, a desire to reduce occupational exposure to pesticides, and minimizing the impact of pesticides on beneficial insect species and the environment. The use of integrated pest management (IPM) can reduce the number of pesticide applications in tree fruit orchards [4,5,6,7]. IPM practices, such as biological monitoring of pests and injury thresholds, and implementation of temperature-driven degree day or weather forecasting models for certain insect pests and diseases, can improve pesticide application efficiency and reduce the unnecessary use of pesticides. However, the low tolerance for damage in tree fruits, especially those marketed for fresh consumption, provides little economic incentive for growers to deviate from current chemically based control measures.

Fruit bagging is a production practice that involves placing bags over developing fruit to exclude pests, reduce chemical residues, and improve overall appearance and quality of fruit [8,9,10,11]. Although fruit bags have been used extensively in Asia as a physical protection method to reduce pest problems in commercial apple orchards [12], few studies examining fruit bagging methods in the United States have been published. Because fruit bagging is labor intensive, it is generally only recommended in the United States as a pest management option in small-scale orchards [13], or in high-value specialty markets [14]. In West Virginia, 85% of apple operations are less than 2 hectares in size [15]. For these small orchards, fruit bagging may be an appropriate non-chemical tactic to incorporate into current IPM programs if bags are easy to set up and maintain during the growing season, and fruit protection from pests is similar to that of conventional chemical controls.

Studies evaluating the practice of fruit bagging outside of Asia have shown reductions in fruit damage from several key apple pests when compared with non-bagged control fruit. In Brazil, bagging fruit with transparent micro-perforated plastic and non-textured fabric bags reduced damage from the South American fruit fly (*Anastrepha fraterculus* [Wiedemann]), Oriental fruit moth (*Grapholita molesta* [Busck]), and Brazilian apple leafroller (*Bonagota salubricola* [Meyrick]), but did not protect fruit from key diseases such as apple scab (*Venturia inaequalis* [Cooke] G. Winter) [16]. In Western United States, bagging fruit with brown paper bags in California [14], and nylon mesh bags in New Mexico [13], significantly reduced fruit damage from codling moth (*Cydia pomonella* [L.]). Despite these findings, it is unclear how successful fruit-bagging would be in West Virginia and other areas of the Northeastern United States where growers must contend with a myriad of key insect pests and diseases throughout the season. Furthermore, it remains uncertain if this management option can provide an equivalent level of fruit protection compared to conventional chemical controls.

The purpose of this study was to evaluate the effectiveness of bagging apples with three types of bagging materials to prevent damage by direct insect pests and diseases and to determine the effectiveness of fruit bagging as a pest management option compared with a conventional pesticide management program.

## 2. Materials and Methods

### 2.1. Study Site

Studies were conducted from 2013 to 2015 in a 1.0 ha research apple orchard located at the West Virginia University Kearneysville Tree Fruit Research and Education Center (WVU-KTFREC) near Kearneysville, WV. The orchard contained ‘Red Delicious’ trees on M.111 rootstock, which measured ~3.7 m in height and width and were planted at a spacing of 4.9 m between trees and 7.3 m between rows. All trees used in the study were planted in 1980. The orchard was under an early season (silver-tip through petal fall) management program for arthropod pests and diseases during each year of the study to protect fruit prior to bagging (Table 1). Minimal fungicide inputs were applied in 2013 because only insect data was collected during that year. All pesticides were applied with a Swanson DA-500A airblast sprayer calibrated to deliver 935 L/ha (100 gpa). 

### 2.2. Fruit Bags

Fruit bags evaluated during the study included two-layer commercial bags and handmade plastic and paper bags. The two-layer commercial bags were similar to those used in the apple industry in Asia. Commercial fruit bags (Wilson Orchard and Vineyard Supply, Union Gap, WA, USA) measured 15.2 cm by 17.8 cm and were made of two separate layers; a waxy plastic inner layer and a medium weight paper outer layer. Bags were secured around individual apples with a thin wire embedded along one edge of the bag. 

Plastic and paper bags were chosen as a low-cost alternative to commercial fruit bags recommended in several home orchard pest management guides [17,18,19]. Plastic bags (Target Corporation, Minneapolis, MN, USA) were made from quart sized zipper locking storage bags measuring 17.7 cm by 19.6 cm. The pull tabs at the top of each plastic bag were removed and the bottom corners cut off diagonally ~5 mm above the corner to prevent the buildup of condensation. Bags were secured around individual apples by centering the apple in the bag and sealing both sides of the zipper lock to the stem. Two staples were placed along the zipper lock on each side of the stem to ensure that the bag remained secured.

Paper bags (Target Corporation, Minneapolis, MN, USA) were made from brown paper lunch bags measuring 13.0 cm by 27.0 cm. The top 8 cm of each paper bag was removed so that the bag dimensions were similar in length to the other fruit bags evaluated. Paper bags were secured to individual apples by cutting a ~3 cm slit in the bottom of the bag and slipping the opening over the fruit. The slit was then pinched together and stapled secure, and the open end of the bag was stapled closed.

### 2.3. Treatments and Experimental Design

In 2013, a preliminary study consisting of two treatments was arranged in a completely randomized design and replicated fifteen times in single tree plots. Treatments included bagging apples with commercial fruit bags and no bagging (untreated control). Within the bagging plots, commercial fruit bags were randomly placed over 20 fruit per plot within each quadrant of treatment trees on June 4. Two weeks before harvest, the location of all bagged fruit was marked, and the bags removed to allow fruit to color properly. 

In 2014 and 2015, five treatments were arranged in a randomized complete block design and replicated five times in single tree plots with at least one buffer tree and one buffer row separating treatment trees. Treatments included a conventional pesticide management program, bagging apples with commercial, plastic, and paper bags, and no bagging (untreated control). Only the conventional pesticide management treatment included post-petal fall applications of pesticides for control of arthropod pests and diseases during the season (Table 2). Within the bagging plots, fruit bags were randomly placed over 50 fruit per plot within each quadrant of treatment trees on 21 May 2014 and 3 June 2015. In 2013, brown marmorated stink bugs invaded trees late in the season after bags were removed, resulting in significant injury to fruit. Bags were therefore left on fruit until harvest in 2014 and 2015 to evaluate their ability to reduce stink bug injury.

### 2.4. Fruit Damage Assessment

All apples assessed in the study were hand thinned to a single fruit per cluster at the time of bagging, which occurred when the average diameter of apples was ~3 cm in size. In the conventional pesticide and control plots, fruit on at least 4 primary scaffold limbs (one in each quadrant) were thinned to a single fruit per cluster. Each limb was then marked so that apples could be identified and randomly selected for later assessment.

Fruit damage was evaluated at harvest in mid-September of each year. All bagged fruit remaining on trees at harvest, as well as 20 fruit (300 total) from the control plots in 2013, and 50 fruit (250 total) from the conventional pesticide and control plots in 2014 and 2015, were assessed. Fruit were classified as free of damage (clean) or damaged by pests. A fruit was considered damaged if it exhibited signs of individual pest infestation or infection. During 2013–2015, fruit damage from the following insect pests were recorded: Brown marmorated stink bugs (*Halyomorpha halys* [Stål]), internal-feeding Lepidoptera species (codling moth, *C. pomonella*; and oriental fruit moth, *G. molesta*), leafroller species (red-banded leafroller, *Argyrotaenia velutinana* [Walker]; tufted apple bud moth, *Platynota idaeusalis* [Walker]; and oblique-banded leafroller, *Choristoneura rosaceana* [Harris]), plum curculio (*Conotrachelus nenuphar* [Herbst]), tarnished plant bug (*Lygus lineolaris* [Palisot de Beauvois]), San Jose scale (*Quadraspidiotus perniciosus* [Comstock]), apple maggot (*Rhagoletis pomonella* [Walsh]), and European apple sawfly (*Hoplocampa testudinea* Klug). During 2014–2015, fruit damage from the following diseases were recorded: Apple scab (*V. inaequalis*), sooty blotch and flyspeck complex, apple fruit rots (*Botryosphaeria obtusa* [Schwein.] Shoemaker and *Botryosphaeria dothidea* [Moug. ex Fr.] Ces. et & de Not.), and cedar apple rust (*Gymnosporangium juniperi-virginianae* Schwein). The number of fruit damaged by pests was converted to proportion damaged by dividing the number damaged by the total number of fruit evaluated from each treatment plot.

### 2.5. Fruit Quality Assessment

In 2015, subsamples of 10 apples were taken from each treatment plot. These apples were evaluated for sugar content and size. Sugar content was measured in % degree Brix by using a handheld refractometer (model AAORHB-32ATC, Ade Advanced Optics, Oregon City, OR, USA), and size was assessed by cutting apples in half and measuring the diameter of fruit at the widest point.

### 2.6. Bagging Set-Up Time and Durability

In 2014 and 2015, the time associated with bagging fruit within each of the fruit bag treatments was recorded. All trees were bagged by a single operator who was equipped with an electronic timer. The operator recorded the time required to thin and bag 50 apples within all quadrants of each treatment tree. At harvest, the number of fruit bags remaining on apples was recorded.

### 2.7. Data Analysis

All data were analyzed separately by year using analysis of variance (PROC GLM) and Tukey’s honestly significant difference (HSD) test (SAS Institute 2008). All proportion data were subjected to arcsine square root transformation prior to analysis to meet the assumptions of normality and homogeneity of variances. Results from all tests were considered significantly different at *p* < 0.05. 

## 3. Results

### 3.1. Fruit Damage

Significant differences in the proportion of clean fruit were found among treatments during each year of the study (Table 3). All treatments provided significantly greater fruit protection from insect pests than the untreated control in 2013 (*F* = 28.61; df = 1,29; *p* ≤ 0.0001), 2014 (*F* = 4.16; df = 8,24; *p* = 0.0074), and in 2015 (*F* = 21.56; df = 8,24; *p* ≤ 0.0001). In addition, all treatments provided significantly greater fruit protection from diseases than the untreated control in 2014 (*F* = 11.05; df = 8,24; *p* = ≤ 0.0001), and 2015 (*F* = 78.21; df = 8,24; *p* ≤ 0.0001). When damage from insects and diseases were combined, all treatments similarly provided significantly greater fruit protection than the untreated control in 2014 (*F* = 7.74; df = 8,24; *p* = 0.0003), and 2015 (*F* = 54.30; df = 8,24; *p* ≤ 0.0001). Although treatment effects were variable each year, plastic bags provided numerically greater fruit protection from insects than all other treatments in 2014 and 2015 (Table 3). The commercial bag and conventional pesticide treatments provided numerically greater fruit protection from diseases than all other treatments in 2014 and 2015 (Table 3). 

The mean proportion of fruit damaged by insects was variable by year. However, the highest levels of fruit damage in untreated plots were consistently caused by the brown marmorated stink bug, internal-feeding Lepidoptera (primarily codling moth), and leafrollers (Table 4). All treatments provided significantly greater fruit protection from brown marmorated stink bug than the untreated control in 2013 (*F* = 5.27; df = 1,29; *p* = 0.0294), 2014 (*F* = 5.69; df = 8,24; *p* = 0.0016), and 2015 (*F* = 14.51; df = 8,24; *p* ≤ 0.0001). All treatments provided significantly greater fruit protection from internal-feeding Lepidoptera than the untreated control in 2013 (*F* = 8.93; df = 1,29; *p* = 0.0058), and 2015 (*F* = 18.60; df = 8,24; *p* ≤ 0.0001). In 2014, all treatments provided significantly greater fruit protection from internal-feeding Lepidoptera than the untreated control (*F* = 4.65; df = 8,24; *p* = 0.0043), except the paper bag treatment, which was not significantly different. Despite the variability of treatment effects each year, plastic bags consistently provided numerically greater fruit protection from brown marmorated stink bug than all other treatments (Table 4). The conventional pesticide treatment consistently provided numerically greater fruit protection from internal-feeding Lepidoptera than all other treatments (Table 4). All treatments provided significantly greater fruit protection from leafrollers than the untreated control in 2013 (*F* = 11.21; df = 1,29; *p* = 0.0023) and 2015 (*F* = 18.36; df = 8,24; *p* ≤ 0.0001), but there were no significant differences among treatments in 2014 (*F* = 1.79; df = 8,24; *p* = 0.1535). Other insects causing sporadic levels of damage included the plum curculio, tarnished plant bug, and San Jose scale (Table 4). Although all treatments provided significantly greater fruit protection from plum curculio and tarnished plant bug than the untreated control in 2015 (plum curculio, *F* = 24.24; df = 8,24; *p* ≤ 0.0001; tarnished plant bug, *F* = 18.43; df = 8,24; *p* ≤ 0.0001), there were no significant differences among treatments in 2013 (plum curculio, *F* = 0.32; df = 1,29; *p* = 0.5742; tarnished plant bug, *F* = 0.29; df = 1,29; *p* = 0.5959) and 2014 (plum curculio, *F* = 0.29; df = 8,24; *p* = 0.9605; tarnished plant bug, *F* = 1.74; df = 8,24; *p* = 0.1657). The mean proportion of fruit damaged by San Jose scale and resulting treatment effects were variable by year. There were no significant differences among treatments in 2013 (*F* = 0.77; df = 1,29; *p* = 0.3881) and 2014 (*F* = 1.95; df = 8,24; *p* = 0.1224). In 2015, the conventional pesticide treatment provided significantly greater fruit protection from the San Jose scale than the paper bag treatment (*F* = 3.25; df = 8,24; *p* = 0.0393), but was similar to all other treatments. Damage from European apple sawfly and apple maggot were negligible during the study and represented less than 1% of injured fruit. 

Although the mean proportion of fruit damaged by recorded diseases was also variable by year, the highest levels of damage were consistently caused by apple scab and sooty blotch and flyspeck complex (Table 5). All treatments provided significantly greater fruit protection from apple scab than the untreated control in 2014 (*F* = 5.31; df = 8,24; *p* = 0.0023), and 2015 (*F* = 9.64; df = 8,24; *p* ≤ 0.0001). All treatments provided significantly greater fruit protection from sooty blotch and flyspeck complex than the untreated control in 2014 (*F* = 16.03; df = 8,24; *p* ≤ 0.0001). In 2015, all treatments provided significantly greater fruit protection from sooty blotch and flyspeck complex than the untreated control (*F* = 39.21; df = 8,24; *p* ≤ 0.0001), except the plastic bag treatment, which was not significantly different. All treatments provided significantly greater fruit protection from fruit rot diseases than the untreated control in 2015 (*F* = 2.90; df = 8,24; *p* = 0.0335), but there were no significant differences among treatments in 2014 (*F* = 1.14; df = 8,24; *p* = 0.3890). Incidences of cedar apple rust were not observed during the study. 

### 3.2. Fruit Quality

**Figure 1:**
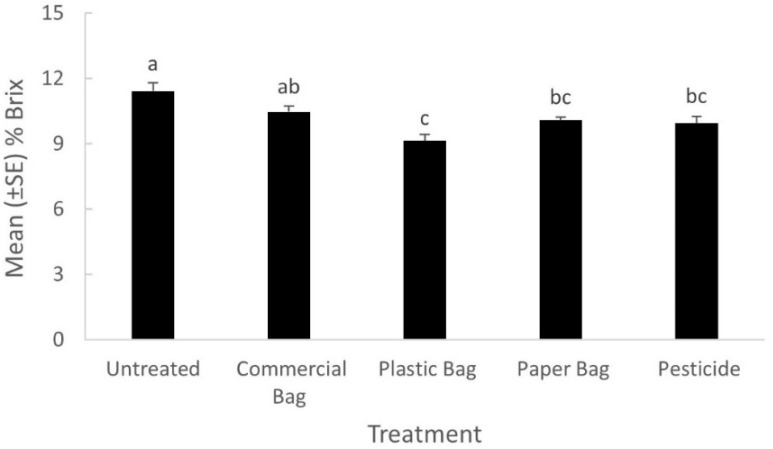
Mean (±SE) percent of Brix of apple fruit in 2015. Bars with different letters denote significant differences among treatments (Tukey’s HSD test, *p* ≤ 0.05).

Fruit quality as measured by percentage Brix was significantly different among treatments (*F* = 4.09; df = 8,24; *p* = 0.0080; Figure 1). The percentage Brix of the apple fruit was significantly higher in the untreated control than all other treatments, except the commercial bag treatment, which was not significantly different. The percentage Brix of apple fruit bagged with plastic was significantly lower than all other treatments except the paper bag and conventional pesticide treatments. Fruit quality as measured by fruit diameter was not significantly different among treatments (*F* = 0.83; df = 8,24; *p* = 0.5921).

### 3.3. Bagging Set-Up Time and Durability

**Figure 2:**
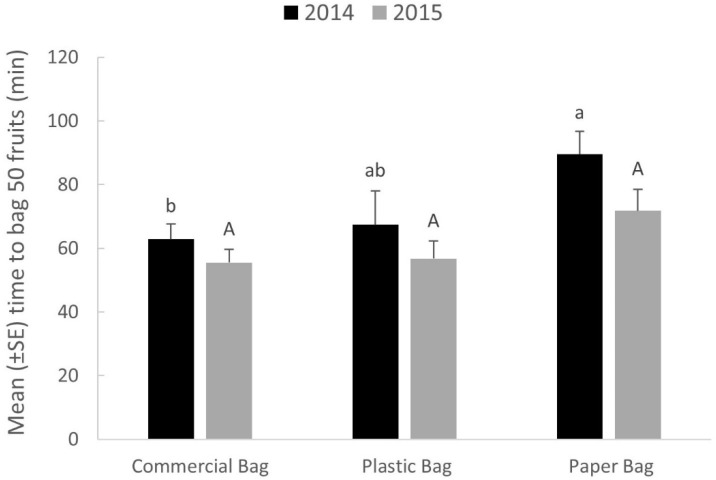
Mean (±SE) effect of bag type on apple bagging times in 2014 and 2015. Bars with different letters denote significant differences among treatments for each year (Tukey’s HSD test, *p* ≤ 0.05).

In 2014, there was a significant effect of bag type on apple bagging times (*F* = 3.88; df = 6,14; *p* = 0.0406; Figure 2). Paper bags required significantly more time to secure around apple fruit than conventional bags. However, there was no significant effect of bag type on apple bagging times in 2015 (*F* = 1.65; df = 6,14; *p* = 0.2502; Figure 2).

The proportion of bags remaining on apples at harvest was significantly different among fruit bagging treatments (Table 6). In 2014, significantly more commercial and plastic bags remained on fruit until harvest than paper bags (*F* = 13.53; df = 6,14; *p* = 0.0008). In 2015, significantly more plastic bags remained on fruit until harvest than all other bag types except commercial bags, which did not differ significantly (*F* = 5.77; df = 6,14; *p* = 0.0135).

## 4. Discussion

Apple orchards in the Northeastern United States are subject to year-to-year fluctuations in rainfall, temperature patterns, and other weather-related phenomena, which can result in variable levels of fruit damage by insects and diseases during a given season. The brown marmorated stink bug, internal-feeding Lepidoptera, leafrollers, apple scab, and sooty blotch/flyspeck consistently caused the greatest amounts of fruit damage in untreated plots. However, several other pests greatly affected fruit quality depending on the year. For instance, tarnished plant bug damage was minor in 2013, but high in 2014 and 2015. Damage from plum curculio was most problematic in 2015, and San Jose scale damage was higher in 2014 than in the other years. Although San Jose scale accounted for the majority of fruit damage in all treatments in 2014 (40% of fruit damage in the conventional pesticide management program), damage to individual apples was relatively minor, with only a small number of scale present near the calyx end of the fruit.

Overall, bagging significantly reduced fruit damage from direct apple pests compared with untreated plots in all three years of the study. For most insect pests and diseases, bagging also provided equivalent levels of fruit protection when compared with the conventional pesticide spray program. Of the three bagging materials evaluated, plastic bags generally provided greater fruit protection from the brown marmorated stink bug, internal-feeding Lepidoptera, tarnished plant bug, and San Jose scale. In fruit bag treatments, damage from insect pests typically occurred where bags failed to cover the fruit as it grew during the season. Plastic bags typically provided better protection over the stem end of the fruit and were less likely to develop tears during the season. These characteristics likely played a substantial role in the lower overall incidence of insect pest damage observed. Two-layered commercial bags generally provided greater fruit protection from diseases. In 2015, fruit bags were placed on apples during a period when sooty blotch/flyspeck outbreaks were highly favored (i.e., above-average summer temperatures combined with frequent rainfall and high humidity), which may have resulted in higher incidence of the disease complex, particularly in the plastic and paper bag treatments.

Previous studies examining fruit bagging of apples showed that various other materials, including nylon mesh, and polypropylene fabric, could be used to protect fruit from certain insect pests [13,16,20] and diseases [20]. Although nylon mesh bags were not examined in this study, the material would be unsuitable for use in areas where piercing/sucking insects such as brown marmorated stink bug are problematic. Personal observations have shown that these insects can easily use their piercing/sucking mouthparts to penetrate the bagging material and feed on fruit. In addition, nylon mesh bags would likely offer little fruit protection from diseases. 

In China and other Asian countries, where fruit bagging is used in apple production, fruit color is enhanced by removing bags and re-exposing fruit to sunlight prior to harvest [21,22]. The coloration of apples during ripening is primarily due to the accumulation of anthocyanins in the fruit peel, which are largely biosynthesized in response to light [23,24]. Apples re-exposed to sunlight following bagging possess higher anthocyanin levels than non-bagged fruit [25], which may account for the greater improvement in fruit color [21]. In 2013, commercial bags were similarly removed from apples two weeks prior to harvest to improve fruit color in the study. However, during this period brown marmorated stink bugs were observed feeding on the previously bagged apples. In 2014 and 2015, all fruit bags were left on apples until harvest to specifically minimize late-season damage from brown marmorated stink bug. Because commercial and paper bags were largely light impermeable, fruit harvested from these treatments were predominately a pale, whitish color. Only fruit from the plastic bag, conventional pesticide management program, and untreated control were properly colored. 

When fruit bags were removed from apples in commercial and paper bag treatments, the European earwig (*Forficula auricularia*) was found in many of the bags at harvest, particularly in 2015. Earwigs are nocturnal, and likely used the light-impermeable bags for shelter; no earwigs were found in plastic bags. Although the European earwig can be a pest of soft-fleshed and injured fruit [26], it is also recognized as an important generalist predator of several apple pests [4,27,28,29]. Apples within the commercial and paper bag treatments did not show signs of feeding damage from earwigs. However, fruit surface areas were often coated with the insect’s frass. It is unclear if the presence of the European earwig in fruit bags had a measurable effect on the incidence of pest damage.

Additional considerations of using fruit bags were the cost and effort in their implementation, as well as their durability over the season. Two-layer commercial fruit bags were $0.14 each, and the total time needed to place bags on 50 fruit ranged from 56–63 min. Plastic bags were a more economical option ($0.04 each), and could be placed on fruit in a similar amount of time (57–67 min to bag 50 fruit). Although paper bags were the cheapest option ($0.01 each), they typically required a greater amount of time to place on fruit (72–90 min to bag 50 fruit). Because of the time and labor needed to place bags on fruit, this pest management option would likely only be suitable in small block orchards. Grasswitz and Fimbres [13] suggest that fruit bagging is a one-time effort, compared with the season-long commitment required in a conventional spray program. However, results from this study suggest that fruit bags would need to be monitored and maintained throughout the season. The proportion of bags that remained on fruit until harvest ranged from 0.54–0.71 (commercial bags), 0.64–0.82 (plastic bags), and 0.32–0.60 (paper bags), depending on the year. Apple orchards in the Northeastern United States are often subject to several strong storms during the growing season. These storms often have high winds, heavy rains, and in some cases hail. During the study, it was common to see bags lying on the orchard floor after such storms.

## 5. Conclusions

In conclusion, this study shows that bagging fruit with two-layer commercial, plastic, and paper bags can reduce the incidence of damage from insect pests and diseases, and can generally provide equivalent levels of fruit protection when compared with a conventional pesticide spray program. However, this pest management technique requires considerably more time to implement, and would not be practical for large-scale apple producers in the United States. Improvements in the methods of securing bags to fruit are needed to make this technique more practical for small-scale apple producers.

## Figures and Tables

**Table 1:** Application date, pesticide, product/formulation, type, and application rate for the early season pest management spray program, 2013–2015.

Year	Application Date	Pesticide	Product/Formulation	Type	Rate/ha(A)
2013	1 April	Copper sulfate	Cuprafix Ultra 40	Fungicide	6.7 kg (6 lb)
		Mineral oil	Damoil	Insecticide	37.4 L (4 gal)
	20 May	Acetamiprid	Assail 30SG	Insecticide	420.3 g (6 oz)
2014	4 March	Copper sulfate	Cuprafix Ultra 40	Fungicide	6.7 kg (6 lb)
		Mineral oil	Damoil	Insecticide	37.4 L (4 gal)
	17 March	Dodine	Syllit 65WG	Fungicide	1.7 kg (1.5 lb)
		Cyprodinil	Vangard 75WG	Fungicide	210.2 g (3 oz)
	24 April	Captan	Captan 80WDG	Fungicide	2.2 kg (2 lb)
		Cyprodinil, Difenoconazole	Inspire Super	Fungicide	876.9 mL (12 fl oz)
	14 May	Mancozeb	Manzate 75DF	Fungicide	3.4 kg (3 lb)
		Fluopyram, Trifloxystrobin	Luna Sensation	Fungicide	423.9 mL (5.8 fl oz)
		Acetamiprid	Assail 30SG	Insecticide	420.3 g (6 oz)
2015	7 April	Copper oxychloride, Copper sulfate	C-O-C-S WDG	Fungicide	11.2 kg (10 lb)
		Mineral oil	BioCover MLT	Insecticide	37.4 L (4 gal)
	15 April	Dodine	Syllit 65WG	Fungicide	1.7 kg (1.5 lb)
		Mancozeb	Manzate 75DF	Fungicide	3.4 kg (3 lb)
		Myclobutanil	Rally 40WSP	Fungicide	280.2 g (4 oz)
		Lambda Cyhalothrin	Warrior II 2CS	Insecticide	182.7 mL (2.5 fl oz)
	22 April	Mancozeb	Manzate 75DF	Fungicide	3.4 kg (3 lb)
		Cyprodinil, Difenoconazole	Inspire Super	Fungicide	876.9 mL (12 fl oz)
	29 April	Fluopyram, Trifloxystrobin	Luna Sensation	Fungicide	365.4 mL (5 fl oz)
		Mancozeb	Manzate 75DF	Fungicide	3.4 kg (3 lb)
		Myclobutanil	Rally 40WSP	Fungicide	280.2 g (4 oz)
	8 May	Mancozeb	Manzate 75DF	Fungicide	3.4 kg (3 lb)
		Cyprodinil, Difenoconazole	Inspire Super	Fungicide	876.9 mL (12 fl oz)
	14 May	Fluopyram, Trifloxystrobin	Luna Sensation	Fungicide	423.9 mL (5.8 fl oz)
		Mancozeb	Manzate 75DF	Fungicide	3.4 kg (3 lb)
		Myclobutanil	Rally 40WSP	Fungicide	350.3 g (5 oz)
		Spinetoram	Delegate 25WG	Insecticide	420.3 g (6 oz)
	26 May	Trifloxystrobin	Flint 50WG	Fungicide	140.1 g (2 oz)
		Mancozeb	Penncozeb 75DF	Fungicide	3.4 kg (3 lb)
		Myclobutanil	Rally 40WSP	Fungicide	350.3 g (5 oz)
		Acetamiprid	Assail 30SG	Insecticide	560.4 g (8 oz)

**Table 2:** Application date, pesticide, product/formulation, type, and application rate for the conventional pesticide management treatment in 2014 and 2015.

Year	Application Date	Pesticide	Product/Formulation	Type	Rate/ha (A)
2014	27 May	Mancozeb	Manzate 75DF	Fungicide	3.4 kg (3 lb)
		Fluopyram, Trifloxystrobin	Luna Sensation	Fungicide	423.9 mL (5.8 fl oz)
		Novaluron	Rimon 0.83EC	Insecticide	1.5 L (20 fl oz)
	10 June	Captan	Captan 80WDG	Fungicide	3.4 kg (3 lb)
		Thiophanate-methyl	Topsin-M 70WSB	Fungicide	840.6 g (12 oz)
		Spinetoram	Delegate 25WG	Insecticide	490.3 g (7 oz)
	24 June	Captan	Captan 80WDG	Fungicide	3.4 kg (3 lb)
		Thiophanate-methyl	Topsin-M 70WSB	Fungicide	840.6 g (12 oz)
		Spinetoram	Delegate 25WG	Insecticide	490.3 g (7 oz)
	8 July	Captan	Captan 80WDG	Fungicide	3.4 kg (3 lb)
		Thiophanate-methyl	Topsin-M 70WSB	Fungicide	840.6 g (12 oz)
		Thiacloprid	Calypso 4F	Insecticide	584.6 mL (8 fl oz)
	22 July	Captan	Captan 80WDG	Fungicide	3.4 kg (3 lb)
		Ziram	Ziram 76DF	Fungicide	4.5 kg (4 lb)
		Methoxyfenozide	Intrepid 2F	Insecticide	1169.2 mL (16 fl oz)
	5 August	Captan	Captan 80WDG	Fungicide	3.4 kg (3 lb)
		Thiophanate-methyl	Topsin-M 70WSB	Fungicide	560.4 g (8 oz)
		Methomyl	Lannate LV	Insecticide	4.2 L (3 pt)
	August 19	Captan	Captan 80WP	Fungicide	3.4 kg (3 lb)
		Methomyl	Lannate LV	Insecticide	4.2 L (3 pt)
2015	25 June	Captan	Captan 80WDG	Fungicide	3.6 kg (3.3 lb)
		Ziram	Ziram 76DF	Fungicide	4.5 kg (4 lb)
		Spinetoram	Delegate 25WG	Insecticide	420.3 g (6 oz)
	July 9	Captan	Captan 80WDG	Fungicide	3.6 kg (3.3 lb)
		Thiophanate-methyl	Topsin-M 70WP	Fungicide	840.6 g (12 oz)
		Ziram	Ziram 76DF	Fungicide	4.5 kg (4 lb)
		Methoxyfenozide	Intrepid 2F	Insecticide	1169.2 mL (16 fl oz)
	30 July	Thiophanate-methyl	Topsin-M 70WP	Fungicide	560.4 g (8 oz)
		Ziram	Ziram 76DF	Fungicide	4.5 kg (4 lb)
		Phosmet	Imidan 70WSB	Insecticide	3.4 kg (3 lb)
	13 August	Thiophanate-methyl	Topsin-M 70WP	Fungicide	560.4 g (8 oz)
		Ziram	Ziram 76DF	Fungicide	4.5 kg (4 lb)
		Phosmet	Imidan 70WSB	Insecticide	3.4 kg (3 lb)
	27 August	Thiophanate-methyl	Topsin-M 70WP	Fungicide	560.4 g (8 oz)
		Ziram	Ziram 76DF	Fungicide	4.5 kg (4 lb)
		Methomyl	Lannate LV	Insecticide	4.2 L (3 pt)

**Table 3:** Mean (±SE) proportion of fruit classified as free of damage.

Year ^1^	Treatment	Proportion Clean from:		
		Insect	Disease	Insect and Disease
2013	Untreated	0.39 ± 0.03 b	*	*
	Commercial Bag	0.68 ± 0.04 a	*	*
2014	Untreated	0.07 ± 0.06 b	0.10 ± 0.05 b	0.01 ± 0.01 b
	Commercial Bag	0.42 ± 0.08 a	0.94 ± 0.03 a	0.41 ± 0.08 a
	Plastic Bag	0.65 ± 0.10 a	0.85 ± 0.11 a	0.63 ± 0.10 a
	Paper Bag	0.53 ± 0.08 a	0.80 ± 0.07 a	0.48 ± 0.09 a
	Pesticide	0.36 ± 0.08 a	0.93 ± 0.02 a	0.32 ± 0.06 a
2015	Untreated	0.14 ± 0.02 b	0.00 ± 0.00 d	0.00 ± 0.00 d
	Commercial Bag	0.68 ± 0.04 a	0.89 ± 0.03 a	0.61 ± 0.01 a
	Plastic Bag	0.84 ± 0.03 a	0.07 ± 0.02 c	0.05 ± 0.02 c
	Paper Bag	0.73 ± 0.05 a	0.31 ± 0.06 b	0.30 ± 0.06 b
	Pesticide	0.74 ± 0.01 a	0.86 ± 0.03 a	0.62 ± 0.04 a

^1^ Within a year, means within a treatment category followed by the same letter are not significantly different, *p* < 0.05, Tukey’s HSD test.

**Table 4:** Mean (±SE) proportion of fruit damaged by insect pests.

Year ^1^	Treatment	BMSB	IL	LR	PC	TPB	SJS
2013	Untreated	0.30 ± 0.04 a	0.09 ± 0.02 a	0.12 ± 0.02 a	0.03 ± 0.01 a	0.01 ± 0.00 a	0.06 ± 0.02 a
	Commercial Bag	0.19 ± 0.04 b	0.02 ± 0.01 b	0.04 ± 0.01 b	0.03 ± 0.02 a	0.01 ± 0.01 a	0.04 ± 0.02 a
2014	Untreated	0.23 ± 0.07 a	0.25 ± 0.06 a	0.11 ± 0.01 a	0.07 ± 0.02 a	0.18 ± 0.03 a	0.30 ± 0.06 a
	Commercial Bag	0.02 ± 0.02 b	0.04 ± 0.01 b	0.05 ± 0.02 a	0.06 ± 0.04 a	0.21 ± 0.09 a	0.43 ± 0.06 a
	Plastic Bag	0.01 ± 0.01 b	0.03 ± 0.02 b	0.07 ± 0.02 a	0.11 ± 0.02 a	0.06 ± 0.02 a	0.13 ± 0.09 a
	Paper Bag	0.02 ± 0.02 b	0.09 ± 0.03 ab	0.07 ± 0.05 a	0.14 ± 0.04 a	0.14 ± 0.08 a	0.23 ± 0.09 a
	Pesticide	0.02 ± 0.01 b	0.00 ± 0.00 b	0.00 ± 0.00 a	0.11 ± 0.04 a	0.07 ± 0.02 a	0.40 ± 0.07 a
2015	Untreated	0.55 ± 0.03 a	0.34 ± 0.02 a	0.22 ± 0.05 a	0.20 ± 0.03 a	0.12 ± 0.03 a	0.04 ± 0.02 ab
	Commercial Bag	0.22 ± 0.04 b	0.08 ± 0.02 b	0.00 ± 0.00 b	0.00 ± 0.00 b	0.00 ± 0.00 b	0.03 ± 0.01 ab
	Plastic Bag	0.03 ± 0.02 c	0.06 ± 0.01 b	0.00 ± 0.00 b	0.02 ± 0.01 b	0.00 ± 0.00 b	0.03 ± 0.01 ab
	Paper Bag	0.13 ± 0.04 b	0.07 ± 0.02 b	0.00 ± 0.00 b	0.00 ± 0.00 b	0.00 ± 0.00 b	0.08 ± 0.03 a
	Pesticide	0.21 ± 0.02 b	0.03 ± 0.01 b	0.02 ± 0.01 b	0.01 ± 0.00 b	0.01 ± 0.01 b	0.00 ± 0.00 b

BMSB, brown marmorated stink bug; IL, internal-feeding Lepidoptera; LR, leafroller; PC, plum curculio; TPB, tarnished plant bug; SJS, San Jose scale. ^1^ Within a year, means within a treatment category followed by the same letter are not significantly different, *p* < 0.05, Tukey’s HSD test.

**Table 5:** Mean (±SE) proportion of fruit damaged by disease.

Year ^1^	Treatment	SB	SBFS	FRD
2014	Untreated	0.32 ± 0.09 a	0.82 ± 0.05 a	0.04 ± 0.02 a
	Commercial Bag	0.01 ± 0.01 b	0.04 ± 0.02 b	0.01 ± 0.01 a
	Plastic Bag	0.06 ± 0.04 b	0.12 ± 0.09 b	0.03 ± 0.02 a
	Paper Bag	0.10 ± 0.08 b	0.14 ± 0.03 b	0.01 ± 0.01 a
	Pesticide	0.06 ± 0.01 b	0.03 ± 0.02 b	0.00 ± 0.00 a
2015	Untreated	0.29 ± 0.04 a	0.96 ± 0.03 a	0.04 ± 0.02 a
	Commercial Bag	0.01 ± 0.01 b	0.10 ± 0.03 c	0.01 ± 0.01 b
	Plastic Bag	0.06 ± 0.02 b	0.90 ± 0.02 a	0.01 ± 0.01 b
	Paper Bag	0.01 ± 0.01 b	0.68 ± 0.05 b	0.00 ± 0.00 b
	Pesticide	0.05 ± 0.02 b	0.10 ± 0.03 c	0.00 ± 0.00 b

SB, apple scab; SBFS, sooty blotch and flyspeck complex; FRD, fruit rot diseases. ^1^ Within a year, means within a treatment category followed by the same letter are not significantly different, *p* < 0.05, Tukey’s HSD test.

**Table 6:** Mean (± SE) proportion of bags remaining on fruit at harvest.

Treatment	Year ^1^	
	2014	2015
Commercial Bag	0.54 ± 0.07 a	0.71 ± 0.04 ab
Plastic Bag	0.64 ± 0.08 a	0.82 ± 0.03 a
Paper Bag	0.32 ± 0.08 b	0.60 ± 0.02 b

^1^ Within a year, means within a treatment category followed by the same letter are not significantly different, *p* < 0.05, Tukey’s HSD test.
